# Overall survival prediction models for gynecological endometrioid adenocarcinoma with squamous differentiation (GE-ASqD) using machine-learning algorithms

**DOI:** 10.1038/s41598-023-33748-1

**Published:** 2023-05-24

**Authors:** Xiangmei Liu, Shuai Jin, Dan Zi

**Affiliations:** 1 Guizhou Medical University, Guiyang, China; 2 School of Big Health, Guizhou Medical University, Guiyang, China; 3 Department of Gynecology and Obstetrics, Guizhou Provincial People’s Hospital, Guiyang, China; 4 Department of Gynecology and Obstetrics, The Affiliated People’s Hospital of Guizhou Medical University, Guiyang, China

**Keywords:** Cancer, Oncology

## Abstract

The actual 5-year survival rates for Gynecological Endometrioid Adenocarcinoma with Squamous Differentiation (GE-ASqD) are rarely reported. The purpose of this study was to evaluate how histological subtypes affected long-term survivors of GE-ASqD (> 5 years). We conducted a retrospective analysis of patients diagnosed GE-ASqD from the Surveillance, Epidemiology, and End Results database (2004–2015). In order to conduct the studies, we employed the chi-square test, univariate cox regression, and multivariate cox proportional hazards model. A total of 1131 patients with GE-ASqD were included in the survival study from 2004 to 2015 after applying the inclusion and exclusion criteria and the sample randomly split into a training set and a test set at a ratio of 7:3. Five machine learning algorithms were trained based on nine clinical variables to predict the 5-year overall survival. The AUC of the training group for the LR, Decision Tree, forest, Gbdt, and gbm algorithms were 0.809, 0.336, 0.841, 0.823, and 0.856 respectively. The AUC of the testing group was 0.779, 0.738, 0.753, 0.767 and 0.734, respectively. The calibration curves confirmed good performance of the five machine learning algorithms. Finally, five algorithms were combined to create a machine learning model that forecasts the 5-year overall survival rate of patients with GE-ASqD.

## Introduction

Gynecologic malignancies are key and more common diseases that affect women's survival^1^. The most frequent histological type of endometrial cancer, endometrioid adenocarcinoma, accounting for 80% of all cases of endometrial cancer^2^. Endometrioid ovarian cancer is a rare epithelial ovarian cancer with pathological features similar to endometrial cancer, accounting for only 10% of epithelial ovarian cancers^3^.

Squamous differentiation is defined as any kind of squamous metaplasia, including morular metaplasia. Typically, endometrioid features include evidence of endometrioid differentiation, including squamous differentiation, complex atypical hyperplasia, and low-grade endometrioid components^4,5^. Studies have revealed that the classification of squamous differentiation components as low-grade or high-grade differentiation is important in order to accurately predict tumor prognosis^6,7^. Squamous differentiation of endometrial cancer has drawn particular attention from researchers because the prognostic variables are still unclear^8^. According to data from a different study, squamous differentiation may operate as a poor prognostic factor in patients with low to moderate endometrioid endometrial cancer by raising the probability of recurrence by a factor of 5.6 times^9^.

Machine learning (ML), an area of artificial intelligence that allows mining the relationships from complex datasets, has been used to make predictions about future outcomes among gynecologic oncology. A deep learning-based automatic staging technique for early endometrial cancer was developed by Mao et al. using MRI scans^10^. According to a multicenter study by Wu et al., an artificial intelligence-based preoperative prediction system could identify and forecast the prognosis of epithelial ovarian cancer^11^. Grimley et al. also utilized a machine learning model to forecast prognostic of patients with epithelial ovarian carcinomas^12^.

Herein, the main purpose of this work was to construct machine learning models and forecast the 5-year survival rate of GE-AsqD patients.

## Methods

### Data collection

The datasets analysed during the current study are available in the SEER databases repository, SEER* Stat 8.3.6, https://seer.cancer.gov/. SEER belong to public databases. It was not necessary to get written informed consent for participating in the present research as the information contained in the SEER database has been de-identified and is publically available following authorization. Users can download relevant data for free for research and publish relevant articles. Our study is based on open source data, so there are no ethical issues and other conflicts of interest.

### Patient and variable selection

**Figure 1:**
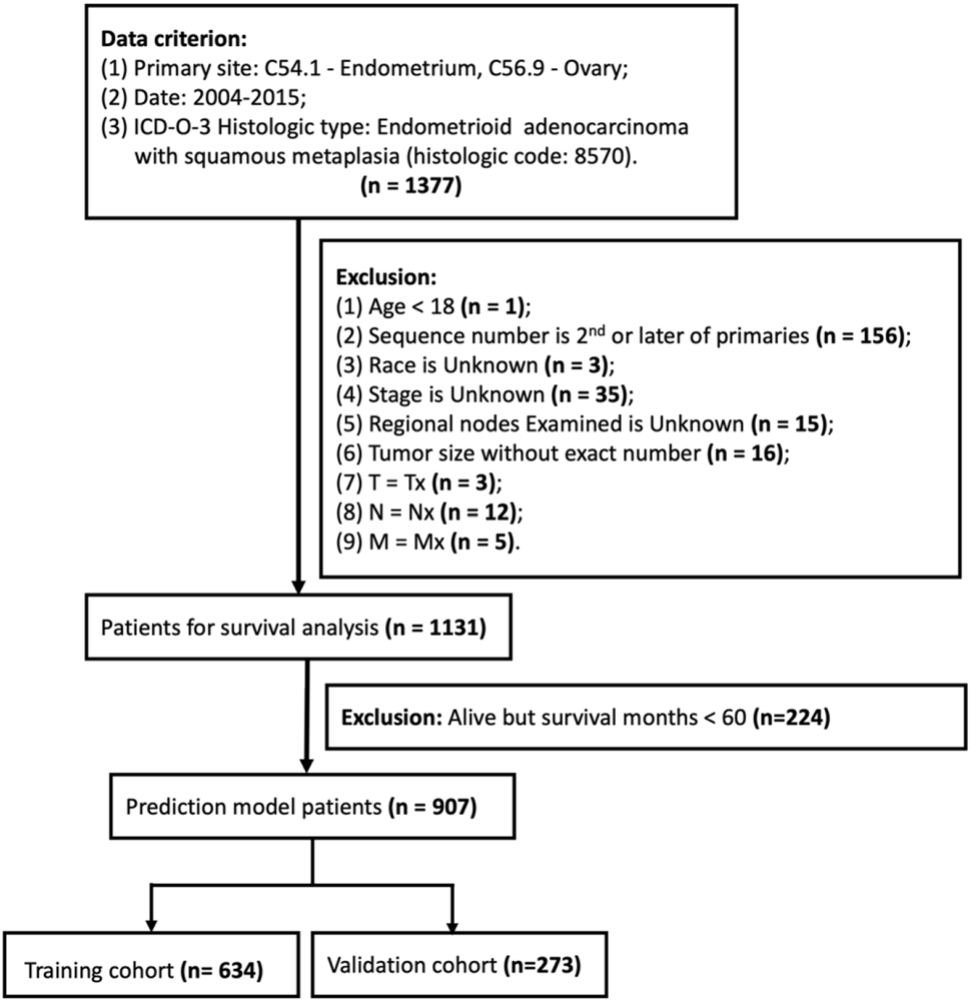
Sample screening process.

We extracted patients diagnosed with gynecological endometrioid adenocarcinoma with squamous differentiation (GE-ASqD) data from the Surveillance, Epidemiology, and End Results (SEER) database. The inclusion criteria were applied: (I) diagnosed between 2004 and 2015; (II) primary site in the endometrium and ovary [International Classification of Diseases for Oncology, third edition (ICD-O-3) code, C54.1, C56.9]; (III) histologically proven malignant carcinosarcoma (ICD-O-3 codes 8570/3). The exclusion criteria were applied: (I) age < 18 year-old, (II) not the primary tumor; (III) unknown information about race, stage, regional nodes examined, tumor size, T, N, M; (IV) For futher training and validation prognostic model analysis, survival time less than 60 months would be excluded. The following clinical pathologic variables were selected: age at diagnosis, race, sequence number, marital status, stage, surgery status, radiation status, chemotherapy status, regional nodes examined (RN Examined), AJCC T, N, M stage, primary site. All patients were staged according to the SEER stage: localized, regional, and distant. We employed the sixth edition of the Derived AJCC Stage Group. It is worth mentioning that the X-tile software (https://medicine.yale.edu/lab/rimm/research/software/) converted continuous variables (age at diagnosis) into categorical variables by determining the optimal cutoff points for each variable^13^. We divided the age at diagnosis into the 18–66, and 67–95-year categories using 66- and 95-year as the cutoff values. The main endpoint was overall survival (OS), which was calculated as the period from diagnosis to death from any cause. The sample was randomly split into a training set and a test set at a ratio of 7:3. The patient selection flowchart is shown in Fig. 1.

### Machine learning models

In this study, we have used several supervised ensemble-based machine learning algorithms, including Logistic Regression (LR), Decision Tree (DT), Random Forest (RF), Light Gradient Boosting Machine (LGBM), and Gradient Boosting (Gbdt) separately to build classification models to stratify GE-ASqD patients., and we searched for the models with the best performance.

In machine learning, a random forest (forest) is a classifier that includes multiple decision trees. The categories of its output are determined by the modes of categories output by individual trees.

The LightGBM (gbm) algorithm is a lifting machine learning algorithm. It is a fast, distributed and high-performing gradient lifting framework based on a decision tree algorithm. It can sort, classify, run regressions, and perform many other machine learning tasks.

The construction of a decision tree model has two steps: induction and pruning. Induction is the step of constructing a decision tree by setting all hierarchical decision boundaries based on data at hand. However, the tree model is subject to severe over-fitting due to the nature of the training decision tree, and this is when pruning is required. Pruning is the process of removing unnecessary branch structures from the decision tree, simplifying the process of overcoming over-fitting and making it easier to interpret.

Elevation is a machine learning technique that can be used for regression and classification problems. It produces a weak prediction model (like a decision tree) at each step and weights it into the total model. If the weak prediction model of each step generates consistent loss function gradient direction, then it is called gradient boosting (Gbdt).

For all machine learning studies, the Python (Python 3.7.13) programming language has been utilized.We have utilized Python libraries such as pandas and numpy for basic data processing and sklearn for machine learning.

The coefficients for the machine learning technique were trained and tested. Evaluation and comparison were completed with the prediction accuracy of a model constructed by machine learning and the area under the curve (AUC). F1-Measure evaluation indicators are used in information retrieval and natural language processing. Precision rate indicates the proportion of correctly classified cases of the sample. Accuracy rate refers to the number of paired cases split by the total number of cases. Recall rate relates to the positive cases in the sample which were predicted correctly. MSE (Mean Squared Error) measures the amount of error in statistical models. Missing data were estimated through multiple imputations.

### Statistical analysis

All statistical analyses were conducted using R version 3.6.1 (www.r-project.org). The association among demographic, clinicopathological, and treatment variables for the histological subtypes was compared using the chi-square test and the Fisher exact test.

Univariate cox regression analysis demonstrated potential prognostic factors with *P* values < 0.1. Multivariate cox proportional hazards model was used to evaluate the prognostic factors associated with OS. Prognostic factors with *P* values < 0.1 on univariate analyses were entered into multivariate analyses. Then, a set of machine learning models were developed base on the independent prognostic factors associated with OS for GE-ASqD patients.

## Results

### Baseline characteristics

After screening data from the SEER database (Fig. 1), we selected a total of 1131 GE-ASqD patients, including 1079 cases of endometrium (EE-ASqD), meanwhile 52 cases of ovary (OE-ASqD). Most of the variables were similarly distributed between EE-AsqD and OE-ASqD. In comparison, approximately 12% and 33% of patients were 1st of 2 or more primaries in the EE-AsqD and OE-AsqD sets, respectively, 75.1% vs. 46.2% as localized, 20.5% vs. 44.2% were regional; radiation (27% vs. 1.9%), chemotherapy (11% vs. 54%) in both sets.The characteristics between the two cancers are shown in detail in Table 1.

**Table 1:** The baseline characteristics of the GE-ASqD patients in SEER database.

Characteristics	Overall (N = 1131)	Endometrium (N = 1079)	Ovary (N = 52)	*p*
Race				0.024
Black	66 (5.8%)	63 (5.8%)	3 (5.8%)	
Other race	108 (9.6%)	97 (9.0%)	11 (21.1%)	
White	957 (84.6%)	919 (85.2%)	38 (73.1%)	
Age				0.056
Elder	226 (20%)	221 (20%)	5 (9.6%)	
Young	905 (80%)	858 (80%)	47 (90.4%)	
Sequence number				< 0.001
1st of 2 or more primaries	145 (13%)	128 (12%)	17 (33%)	
One primary only	986 (87%)	951 (88%)	35 (67%)	
Marital				0.614
Married	604 (53%)	578 (54%)	26 (50%)	
Other Marital	527 (47%)	501 (46%)	26 (50%)	
Stage				< 0.001
Distant	52 (4.6%)	47 (4.4%)	5 (9.6%)	
Localized	835 (73.8%)	811 (75.1%)	24 (46.2%)	
Regional	244 (21.6%)	221 (20.5%)	23 (44.2%)	
Surgery				0.399
None	34 (3.0%)	34 (3.2%)	0 (0%)	
Yes	1097 (97%)	1045 (96.8%)	52 (100%)	
Radiation				< 0.001
No Radiation	837 (74%)	786 (73%)	51 (98.1%)	
Radiation	294 (26%)	293 (27%)	1 (1.9%)	
Chemotherapy				< 0.001
No/Unknown	979 (87%)	955 (89%)	24 (46%)	
Yes	152 (13%)	124 (11%)	28 (54%)	
RN Examined				0.282
No Examined	428 (38%)	412 (38%)	16 (31%)	
Yes Examined	703 (62%)	667 (62%)	36 (69%)	
T				0.732
T1	895 (79.1%)	854 (79.2%)	41 (78.8%)	
T2	112 (9.9%)	105 (9.7%)	7 (13.5%)	
T3	115 (10.2%)	111 (10.3%)	4 (7.7%)	
T4	9 (0.8%)	9 (0.8%)	0 (0%)	
N				0.222
N0	1027 (90.8%)	977 (90.5%)	50 (96.2%)	
N1	104 (9.2%)	102 (9.5%)	2 (3.8%)	
M				> 0.999
M0	1085 (95.9%)	1035 (95.9%)	50 (96.2%)	
M1	46 (4.1%)	44 (4.1%)	2 (3.8%)	

According to the results of univariate cox regression analysis, we found that race, age at diagnosis, sequence number, stage, surgery status, radiation status, chemotherapy status, regional nodes, T, N, M stage were potentially correlated with the OS of GE-ASqD (*P* < 0.05).

These potential prognostic factors were evaluated through multivariate regression analysis, which indicated that race [other race vs. black : hazard ratio (HR) = 0.42, 95% confidence interval (CI) 0.21–0.085, *P* = 0.016 ], age at diagnosis [18–66 years vs. 67–95 years: hazard ratio (HR) = 0.27, 95% confidence interval (CI) 0.21–0.35, *P* < 0.001], sequence number[1st of 2 or more primaries vs. 1st of 2 or more primaries: hazard ratio (HR) = 0.58, 95% confidence interval (CI) 0.41–0.8, *P* = 0.001], surgical status (yes vs. none : HR = 0.28, 95% CI 0.16–0.49, *P* < 0.001), radiation status (radiation vs. no radiation: HR = 1.44, 95% CI 1.07–1.94, *P* = 0.016), chemotherapy status (chemotherapy vs. no/unknown: HR = 0.6, 95% CI 0.4–0.91, *P* = 0.016), RN Examined (yes vs. no : HR = 0.54, 95% CI 0.4–0.73, *P* < 0.001), T stage (T3 vs. T1 : HR = 3.45, 95% CI 2.32–5.12, *P* < 0.001), N stage (N1 vs. N0 : HR = 2.9, 95% CI 1.91–4.39, *P* < 0.001), and M stage (M1 vs. M0: HR = 3.03, 95% CI 1.81–5.01, *P* < 0.001) were independent prognostic factors for GE-ASqD (P < 0.05). The results of the univariate and multivariate cox regression analysis are listed in detail in Table 2.

**Table 2:** The baseline characteristics, univariate and multivariate cox analysis.

Characteristics	Univariate Cox		Multivariate Cox	
	HR (95% CI)	*p*	HR (95% CI)	*p*
Race				
Black	Reference		Reference	
Other race	0.49 (0.25–0.97)	0.039	0.42 (0.21–0.85 )	0.016
White	0.71 (0.44–1.14)	0.156	0.73 (0.44–1.2 )	0.214
Age				
Elder	Reference		Reference	
Young	0.28 (0.22–0.37)	< 0.001	0.27 (0.21–0.35 )	< 0.001
Sequence number				
1st of 2 or more primaries	Reference		Reference	
One primary only	0.64 (0.46–0.89)	0.007	0.58 (0.41–0.8 )	0.001
Marital				
Married	Reference		Reference	
Other marital	1.25 (0.96–1.62)	0.091	–	–
Stage				
Distant	Reference		Reference	
Localized	0.11 (0.08–0.17)	< 0.001	–	–
Regional	0.32 (0.21–0.48)	< 0.001	–	–
Surgery				
None	Reference		Reference	
Yes	0.31 (0.19–0.51)	< 0.001	0.28 ( 0.16–0.49 )	< 0.001
Radiation				
No radiation	Reference		Reference	
Radiation	1.96 (1.5–2.56)	< 0.001	1.44 (1.07–1.94 )	0.016
Chemotherapy				
No/Unknown	Reference		Reference	
Yes	2.03 (1.48–2.79)	< 0.001	0.6 ( 0.4–0.91 )	0.016
RN examined				
No examined	Reference		Reference	
Yes examined	0.84 (0.64–1.09)	0.179	0.54 ( 0.4–0.73 )	< 0.001
T				
T1	Reference		Reference	
T2	2.76 (1.94–3.94)	< 0.001	2.67 (1.83–3.91)	< 0.001
T3	4.39 (3.19–6.02)	< 0.001	3.45 (2.32–5.12 )	< 0.001
T4	7.57 (3.34–17.18)	< 0.001	1.82 (0.71–4.69 )	0.214
N				
N0	Reference		Reference	
N1	3.97 (2.91–5.41)	< 0.001	2.9 (1.91–4.39 )	< 0.001
M				
M0	Reference		Reference	
M1	6.44 (4.41–9.42)	< 0.001	3.03 (1.83–5.01 )	< 0.001
Primary site				
Endometrium	Reference		Reference	
Ovary	0.79 (0.39–1.6)	0.509	–	-

Alive but survival months < 60 months were excluded, finally 907 patients remain for further analysis. Table 3 summarizes the baseline characteristics of the training and validation sets. All variables were similarly distributed between the two sets, with EE-ASqD (95.9% vs. 94.9%) and OE-ASqD (4.1% vs. 5.1%) in the training and validation sets. In both sets, almost all patients sequence number were the one primary only (86% vs. 88%). Most of the patients in the training and validation sets were white (83.8% vs. 84.3%), 18–66-year (78% vs. 81%), and married (51% vs. 58%). The clinical data demonstrated a relatively localized (71.6% vs. 75.8%) malignancy; In comparison, approximately 77% and 80.2% of patients in the training and validation sets, respectively, was T1 stage, 89% vs.92.7% of N0, and 95% vs. 96% as M0. In both sets, almost all patients received surgery (96.5% vs. 96.3%), for regional nodes examination (RN Examined) were done (63% vs. 61%) in the training and validation sets furthermore. whereas only a few patients received chemotherapy (13%) and radiation (26% vs. 23%) in the training and validation sets.

**Table 3:** The baseline characteristics of the training and validation sets used in the prognostic model.

Characteristics	Training (N = 634) n (%)	Validation (N = 273) n (%)	*P*
Race			0.973
Black	42 (6.6%)	17 (6.2%)	
Other race	61 (9.6%)	26 (9.5%)	
White	531 (83.8%)	230 (84.3%)	
Age			0.384
Elder	137 (22%)	52 (19%)	
Young	497 (78%)	221 (81%)	
Sequence number			0.430
1st of 2 or more primaries	89 (14%)	33 (12%)	
One primary only	545 (86%)	240 (88%)	
Marital			0.050
Married	322 (51%)	158 (58%)	
Other marital	312 (49%)	115 (42%)	
Stage			0.420
Distant	34 (5.4%)	13 (4.8%)	
Localized	454 (71.6%)	207 (75.8%)	
Regional	146 (23%)	53 (19.4%)	
Surgery			0.885
None	22 (3.5%)	10 (3.7%)	
Yes	612 (96.5%)	263 (96.3%)	
Radiation			0.358
No Radiation	467 (74%)	209 (77%)	
Radiation	167 (26%)	64 (23%)	
Chemotherapy			0.811
No/Unknown	549 (87%)	238 (87%)	
Yes	85 (13%)	35 (13%)	
RN examined			0.615
No examined	235 (37%)	106 (39%)	
Yes examined	399 (63%)	167 (61%)	
T			0.301
T1	488 (77%)	219 (80.2%)	
T2	69 (10.9%)	27 (9.9%)	
T3	73 (11.5%)	23 (8.4%)	
T4	4 (0.6%)	4 (1.5%)	
N			0.113
N0	566 (89%)	253 (92.7%)	
N1	68 (11%)	20 (7.3%)	
M			0.508
M0	602 (95%)	262 (96%)	
M1	32 (5.0%)	11 (4.0%)	
Primary site			0.489
Endometrium	608 (95.9%)	259 (94.9%)	
Ovary	26 (4.1%)	14 (5.1%)	

### Prognostic model construction and model performance

**Figure 2:**
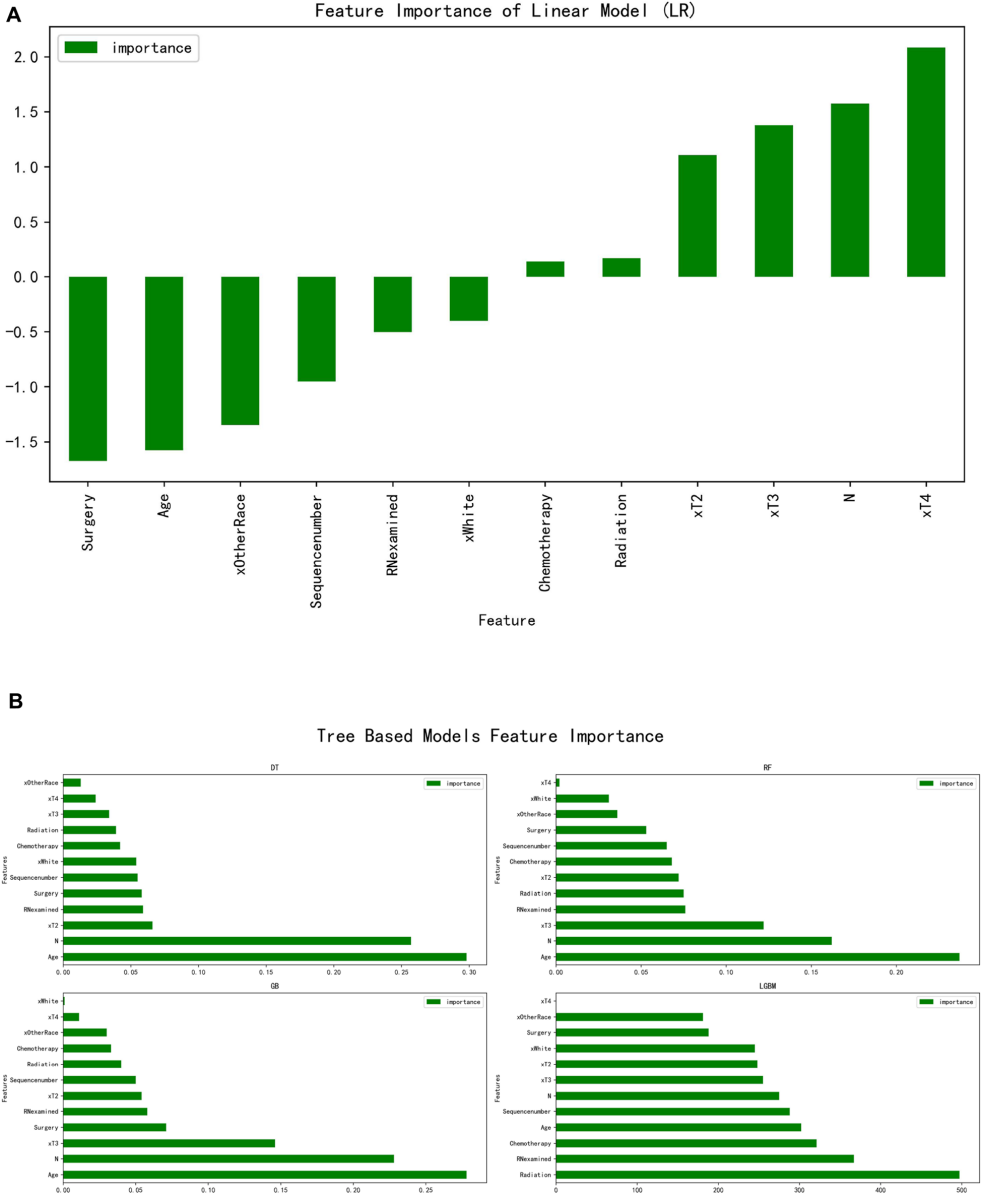
(**A**) The Linear model was used to calculate the importance of each feature. The bar chart depicts the relative significance of the variables. (**B**) The tree-based model was used to calculate the importance of each feature. The bar chart depicts the relative significance of the variables.

In this study, the dataset consisted of 907 individual patients’ information. We divided the whole dataset into 70% for training and 30% for testing. Accuracy, Precision, Recall, F1-score, AUC, and MSE evaluation metrics were employed to test the classifier performance. Figure 2A shows the associated independent risk factors based on a multiple linear regression model. Multiple linear regression models are used to quantify the relationship between predictor variables and a response variable takes on a continuous value. Two of the most important values in a regression table are the regression coefficients and their corresponding p-values. The p-values inform whether or not there is a statistically significant relationship between each predictor variable and the response variable. Because the output of a linear regression model is continuous value. It is possible to get negative values as well as the output. It is different from logistic regression model, which returns probability as the output varies between 0 and 1. The most significant descending order parameters were age at diagnosis, N stage, T2 stage, RN Examined, and surgery status in Fig. 2B of DT model. The age at diagnosis, N stage, T3 stage, RN Examined, and radiation status are the most critical attribute in descending order in the RF model. The highest vital features in descending order are age at diagnosis, N stage, T3 stage, surgery and RN Examined, status in the GB model. The radiation, RN Examined, chemotherapy status, age at diagnosis and sequence number are the most critical attribute in descending order in the LGBM model.

**Figure 3:**
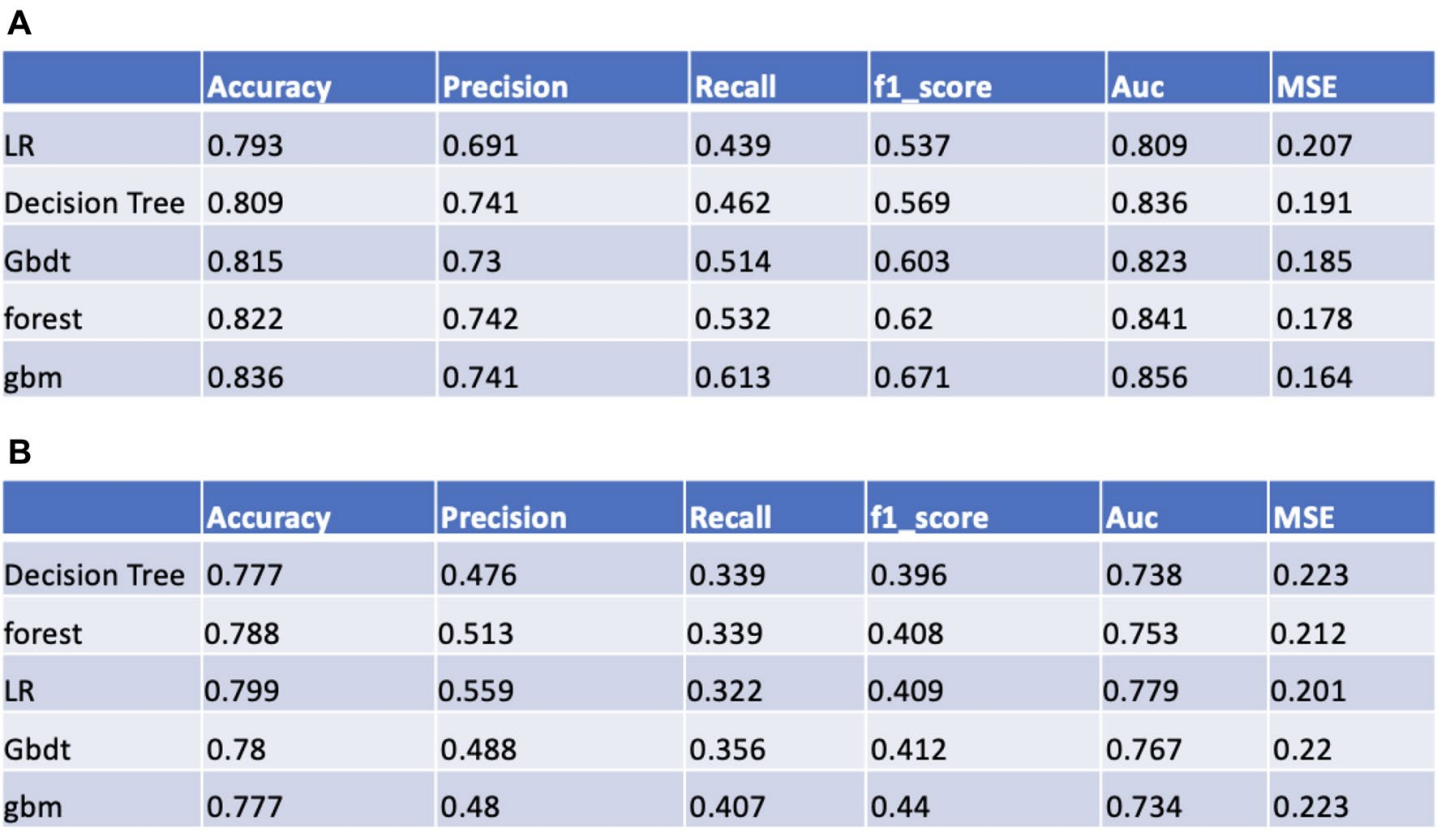
(**A**) Forecast results of train group. (**B**) Forecast results of testing group.

**Figure 4:**
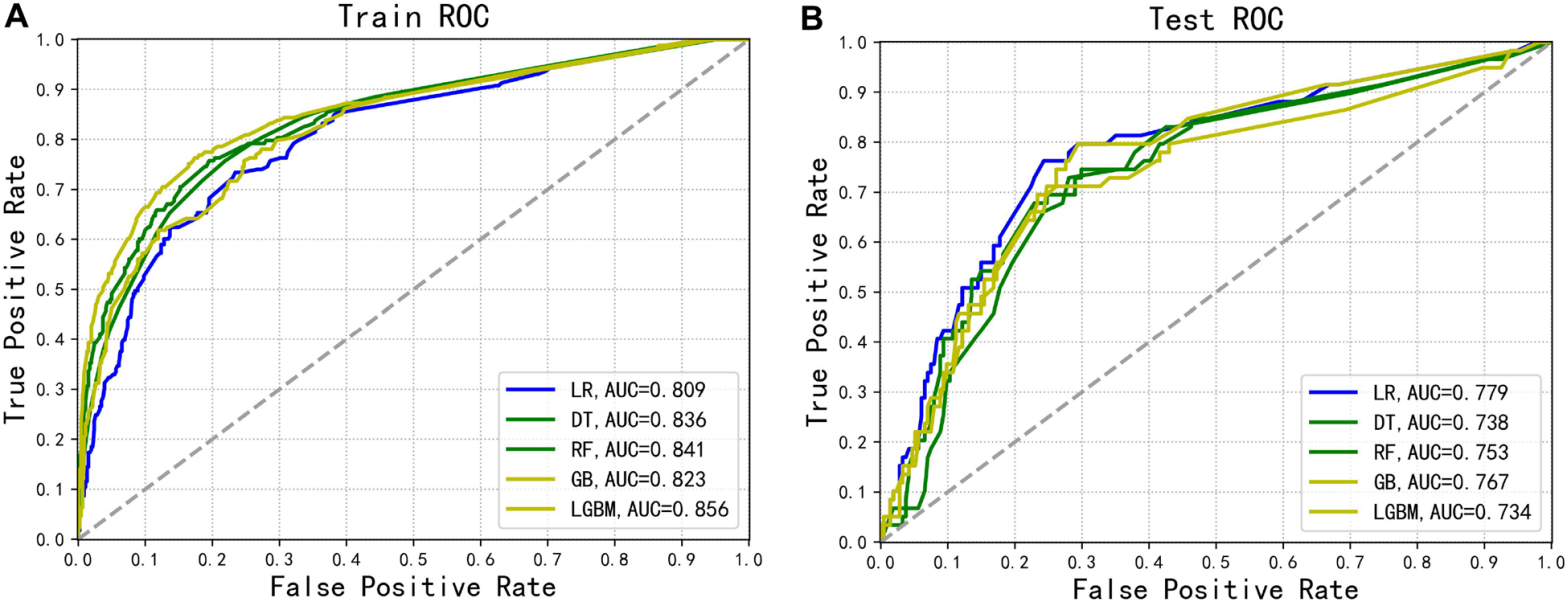
(**A**) ROC curves of the models for the training cohorts. (**B**) ROC curves of the models for the testing cohorts.

As show in Figs. 3A and 4A, the models constructed by the five machine learning algorithms in the training group are compared. Among the five machine learning algorithms, gbm and random forest have the highest accuracy, 0.836 and 0.822 respectively. The highest precision of the five algorithms was random forest, 0.742. The highest recall rate was that of the gbm algorithm (0.613). Among the five algorithms, gbm had the highest accuracy, recall rate and f1 score, and Auc, 0.836, 0.613, 0.671 and 0.856, respectively. The AUC values for the four algorithms were: gbm (0.856), forest (0.841), DecisionTree (0.836). Gbdt (0.823) and LR (0.809). Among the five algorithms, gbm had the lowest MSE value (0.164).

**Figure 5:**
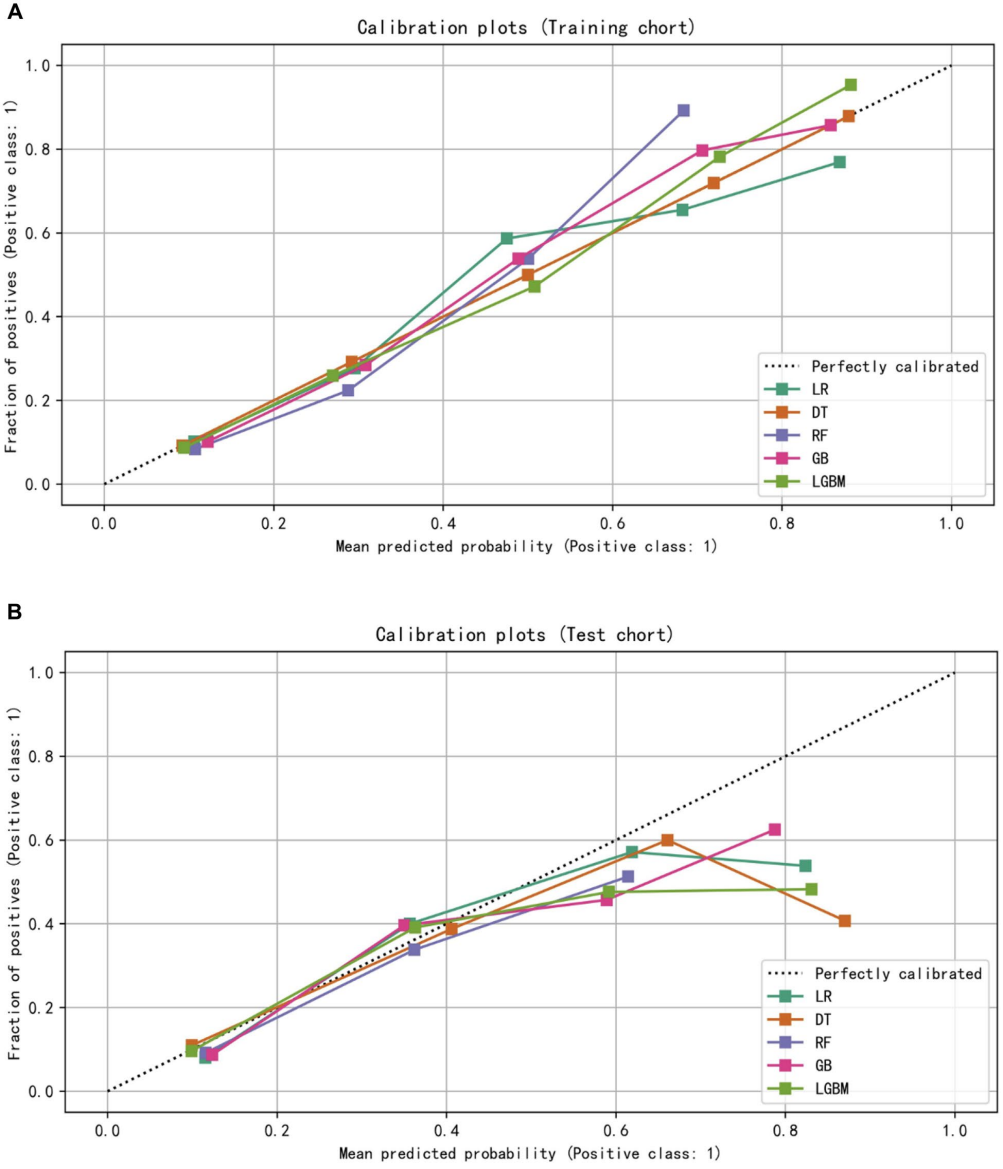
(**A**) The calibration plots for predict 5-year GE-AsqD OS for the training and testing set. (**B**) The calibration plots for predict 5-year GE-AsqD OS for the training and testing set.

The models constructed by four machine learning algorithms in the test group are compared (Figs. 3B and 4B). LR had the highest accuracy (0.799), precision (0.559) and Auc (0.779). The recall rate and f1-score for the gbm algorithm was 0.407 and 0.44. The lowest f1 score was that of decision tree at 0.059. The AUC values of the five algorithms were: LR (0.779), Gbdt (0.767), forest (0.753), DecisionTree (0.738) and gbm(0.734). Among the five algorithms, LR had the lowest MSE value at 0.201. The calibration curves confirm good performance of the five machine learning algorithms (Fig. 5A and 5B).

## Discussion

In the era of “personalized medicine,” the use of prediction models has gained increasing interest among clinicians to guide treatment planning, individualized treatment aims to minimize unnecessary exposure to therapy-related morbidity and at the same time offer proper management for high-risk patients. The combination of PARP inhibitors and immune checkpoint inhibitors considerably improves the prognosis of gynecologic cancer patients and promotes the long-term benefits following maintenance therapy in the era of personalized precision medicine where targeted and immunotherapy are common^14^. Furthermore, Zhang et al. and Chen et al. demonstrated the predictive importance of PD-L1 expression in endometrial serous cancer^15,16^.

It is now evident that OC and EC are not single disease, but is a category comprised of several distinct histotypes. The medical community’s understanding of OC and EC has changed significantly over the past few years^17,18^. Katelyn et al. also suggest that each morphologic subtype has potential therapeutic implications^19^. Additionally, the likelihood of passing away varied noticeably among those with various histological subtypes^20^. Less research has been focused on gynecologic endometrial adenocarcinoma with histological subtypes of squamous differentiation in gynecologic oncology, but this needs to be examined because of the unique prognostic determinant and association pattern our findings suggest throughout the survival trajectory. Data from the SEER program provides a unique opportunity to study a rare disease given the large, nationally-representative sample of cancer patients, extensive follow-up information, and availability of detailed histomorphologic data. According to the data extracted from the SEER database in our current study, GE-ASqD is more common among young whites, generally in the early stages of the disease, and most patients underwent surgical treatment and intraoperative lymph node examination.

Although the demographic characteristics of the present study suggest that non-white populations account for a very small proportion of GE-ASqD, there are many studies on ovarian cancer that suggest poor survival for black compared with other ethnic groups. Thus, the recently established African ancestry women's ovarian cancer (OCWAA) consortium^21^ analyzed key differential factors by analyzing various characteristics of patients in their large national cancer databases or medical databases. Studies have also noted worse survival in black patients with endometrial cancer (EC) compared with white patients, and higher staging and grading, histological risk, and worse survival in black women^22,23^. Studies have also shown that black women are 2.5 times more likely to die from endometrial cancer^24^. In the multivariate analysis of our study, age, surgery, chemoradiotherapy, lymph node examination, and the presence or absence of node-positive metastases were statistically significantly different from patient prognosis analysis. Our analysis based on LR models and tree models showed that T stage and presence of positive lymph node metastasis were significantly associated with 5-year survival. Especially in tree-based models, age and Nodal properties have been shown to be significantly associated with disease survival.

According to international guidelines^25,26^, the fundamental management of gynecological oncology is achieved through standard surgery or cytoreductive surgery performed by a team of trained gynecological oncologists. Most patients present with early-stage disease are cured by surgery. In this study, we did not further detail the classification of surgical procedures for patients, but the results of this study suggest that surgery is an important determinant of patient survival. In the current study, N was assessed as a very important and meaningful clinical variable factor in each model for risk assessment and prognostic prediction, and the model suggested the importance of N intraoperative assessment. Many previous studies and clinical guidelines have indicated that Positivity for pelvic and/or paraaortic lymph node metastasis (LNM) is an important aspect of predicting a worse prognostic outcome, and current guidelines of the National Comprehensive Cancer Network (NCCN) recommend observation for patients with substage IA or IB, grade 1 EEOC^27–29^. The significance of conventional lymphadenectomy for improving outcomes in early clinical endometrial cancer is controversial, but it is strongly associated with a 15% to 20% surgically related morbidity^30^. Few attempts have been made to predict the risk of LNM before surgical treatment^31–33^. In recent years, gynecologic oncologists have chosen to use dye-injected tracer in the first few minutes of the operation, combining with Fluorescence microscope to accurately assess whether there are first-stop Sentinel lymph node and distant lymph node metastases, when the results were positive, the decision was made to perform systemic lymph node dissection and para-aortic lymph node dissection to improve the patient's prognosis^34^. At the same time, the implementation of this technology has also had a positive impact on reducing healthcare costs. Among the patients in this study, ovarian cancer patients receiving radiotherapy and endometrioid carcinoma patients receiving chemotherapy were a minority. It has been demonstrated that radiation therapy increases survival in individuals with high risk of endometrial cancer but not in those with intermediate risk^35^. It also increases costs and a higher risk of morbidity. It is advised that patients with mild endometrial cancer refrain from radiation therapy for the time being, and that they instead undergo follow-up observation. The study points out that by dividing gynecologic tumors into clinically meaningful subgroups, we can better understand the pathological development and pathogenesis of tumors, thus adapting to the era of individualized and precise treatment, to select and improve individualized treatment based on machine learning and other prognostic scientific prediction.

However, there are some limitations to our study. Firstly, SEER lacks data on chemotherapy treatments and patterns and timing of recurrence. Second, outcome data for individuals receiving targeted therapies were not included in the sample, which may have made the prediction model less comprehensive. Finally, it should be admitted that further external validation in different geographic regions and etiology is of necessity.

## Conclusions

In this work, five machine learning algorithms were used to build predictive models after analyzing the clinical traits and prognosis of patients with GE-ASqD. The 5-year OS of patients with GE-ASqD could be accurately predicted using the machine learning model, which may aid clinicians in making more precise and individualized therapeutic decisions. This is especially crucial to boost the long-term prognosis of high-risk patients with histological subtype squamous differentiation.

### Condensation

Machine Learning Algorithm Predictive Model for the 5-year OS of GE-ASqD.

## Data Availability

The code to perform all presented studies is written in R or python and is freely available on GitHub: https://github.com/users/mimimay-cpu/projects/3/views/1?pane=issue&itemId=24276328.
